# Tunable Triple-Band Terahertz Perfect Absorber and Four-Input AND Gate Based on a Graphene Metamaterial

**DOI:** 10.3390/nano16080494

**Published:** 2026-04-21

**Authors:** Shuxin Xu, Lili Zeng, Zhengzheng Shao, Boxun Li, Wenjie Hu, Yiyu Tu, Xingyi Zhu

**Affiliations:** 1 School of Physics and Optoelectronics, Xiangtan University, Xiangtan 411105, China; 202305710908@smail.xtu.edu.cn (S.X.);; 2 New Energy Institute, Hunan Vocational Institute of Technology, Xiangtan 411104, China; 3 School of Physics, Central South University, Changsha 410083, China; 4 Hunan Engineering Laboratory for Microelectronics, Optoelectronics and System on a Chip, Xiangtan 411105, China

**Keywords:** multi-peak, absorber, graphene, fermi level, AND logic gate

## Abstract

This study introduces a switchable and tunable multimodal, multi-peak, perfect terahertz absorber, utilizing a composite structure of graphene and double concentric metal rings. From bottom to top, the absorber consists of a gold substrate, a SiO_2_ dielectric layer, a patterned graphene layer, another SiO_2_ dielectric layer, and double concentric metal rings on the top. The structure achieves three high-absorption resonance peaks in the far-infrared band: a relatively broad peak with 99.05% absorptance at 38.128 THz, and two extremely narrow peaks with 99.56% and 97.23% absorptance at 47.909 THz and 49.873 THz, respectively. Analysis of the absorption spectra and electric field distributions reveals that the generation mechanism of Peak I is Fabry–Pérot cavity resonance, while Peaks II and III result from the coupling between the high-order localized surface plasmons in the outer ring and the graphene surface plasmon polaritons. Benefiting from graphene’s excellent electrical tunability, the absorption peaks’ positions and intensities can be dynamically tuned by varying the Fermi level. The core innovation of this work lies in the high-level integration of multiple functionalities. By leveraging the sensitive response of Peak III to variations in the Fermi level, a four-input AND logic gate is embedded within the metamaterial absorber in this frequency band. The Fermi levels of four independent graphene regions serve as the binary inputs, while the absorption state of Peak III is defined as the logical output. Additionally, the two narrow peaks display high sensitivity to the surrounding refractive index, with sensitivities of 30.1 THz/RIU and 62.5 THz/RIU, demonstrating significant potential for sensing. This multifunctional integrated device combines tunable absorption, a logic gate, and sensing capabilities, making it promising for terahertz communication systems, intelligent sensing networks, and reconfigurable platforms.

## 1. Introduction

The far-infrared band is also regarded as the high-frequency end of terahertz waves. In recent years, electromagnetic waves in the far-infrared band have been widely applied in radar communications, energy harvesting and thermal management, environmental sensing and monitoring, and electromagnetic stealth technology [1,2,3]. Terahertz waves have gained extensive attention in academia due to their enormous application potential. Subsequently, perfect absorbers [4,5,6,7,8], sensors [9,10,11], and optical switches [12,13,14] based on terahertz waves have been broadly researched. As one of the research directions in this field, the perfect absorber can effectively capture and dissipate the energy of incident electromagnetic waves, playing a vital role in applications such as solar thermal receivers, energy harvesting, infrared radiation detection, environmental sensing, and electromagnetic stealth [15,16,17]. Traditional absorbers have major limitations. Once their structure is fabricated, it cannot be changed, resulting in invariable performance [18,19,20]. Graphene is a unique two-dimensional material, composed of carbon atoms arranged in a hexagonal honeycomb lattice. Its exceptional properties arise from the sp^2^ hybridization of carbon atoms and the confinement of electrons within the two-dimensional plane [21]. As a unique two-dimensional material, its Fermi level can be altered by exerting a bias voltage or doping chemically [22,23,24]. By flexibly adjusting the surface plasmon resonance [25] frequency supported by graphene, tunability of the absorber can be achieved. Additionally, the combination of graphene with metallic microstructures can generate abundant resonance modes and coupling effects, providing an innovative way to achieve multi-band absorption, and, due to its extraordinary tunable property, graphene is used in the fabrication of perfect absorbers. Many researchers are dedicated to developing absorbers with higher absorption rates and more absorption peaks [26,27], achieving tunable absorbers including broadband and narrowband absorbers, as well as single-frequency and multi-frequency absorbers [28]. However, the majority of researchers have focused on realizing dynamic tuning of absorption peaks [29,30,31,32]. Currently, the deep integration of multiple functionalities, such as multi-peak absorption, electrically controlled logic operations [33,34,35], and high-sensitivity sensing [36,37], into a single metamaterial structure remains a challenge. Developing an intrinsic photonic device that physically integrates sensing, modulation, and logic functionalities holds significant scientific importance and application value. We aim to go beyond traditional, single-function tunable metamaterials and explore, within a single terahertz metamaterial structure, the possibility of making its optical response programmable by multiple independent electrical signals and directly mappable to discrete logical outputs through ingenious electromagnetic design. This mechanism of “optical absorption state as logical output” provides a new device prototype for realizing high-speed, low-power, terahertz-band photonic logic and potential sensing–computing integration applications.

This paper outlines the design and investigation of a composite metamaterial absorber based on graphene and double concentric metal rings. The absorber achieves three perfect absorption peaks in the terahertz band and exhibits extraordinary tunable characteristics. We deeply probed into the generation mechanisms of these three peaks and found that Peak I arises because the upper metal ring and graphene, together with the bottom gold substrate, form a subwavelength Fabry–Pérot resonant cavity. Electromagnetic waves undergo multiple reflections and interference between the two metal layers. In contrast, Peak II and Peak III are generated by the coupling effect between metal ring plasmon and graphene surface plasmon polaritons. Specifically, Peak II originates from the weak coupling between the high-order localized surface plasmon mode of the outer ring and graphene, while Peak III is dominated by the strong coupling between the localized surface plasmon of the inner ring and graphene surface plasmon polaritons. We further present its application potential in tunable perfect absorbers, in the form of a four-input AND gate with electrically controlled logic operations and high-sensitivity sensors. Meanwhile, the structure possesses polarization insensitivity and wide-incident-angle stability [38]. Developing an intrinsic photonic device that physically integrates sensing [39], modulation, and logic functionalities holds significant scientific importance and application value. We aim to go beyond traditional, single-function tunable metamaterials and explore, within a single terahertz metamaterial structure, the possibility of making its optical response programmable by multiple independent electrical signals and directly mappable to discrete logical outputs through ingenious electromagnetic design. This mechanism of “optical absorption state as logical output” provides a new device prototype for realizing high-speed, low-power, terahertz-band photonic logic and potential sensing–computing integration applications.

## 2. Structural Design and Numerical Model

### 2.1. Structural Design

**Figure 1:**
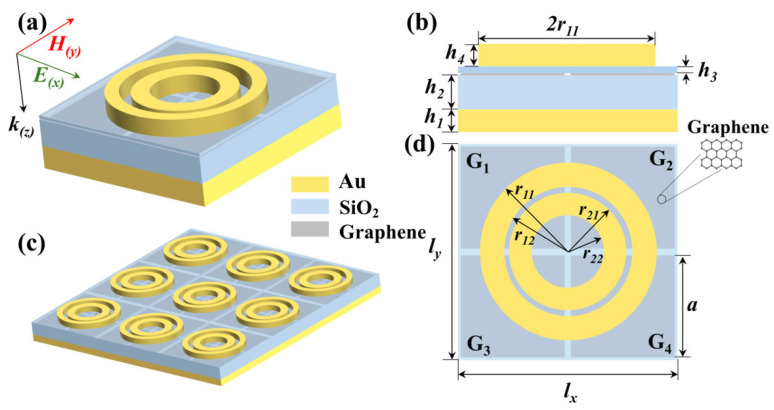
(**a**) Unit diagram of graphene absorber. (**b**) Lateral view of graphene absorber. (**c**) 3D array diagram of graphene absorber. (**d**) Planform of graphene absorber. The structural parameters are as follows: *h*_1_ = 0.2 μm, *h*_2_ = 0.3 μm, *h*_3_ = 0.02 μm, *h*_4_ = 0.2 μm, *l_x_* = *l_y_* = 6 μm, *a* = 2.9 μm, *r*_11_ = 2.4 μm, *r*_12_ = 1.8 μm, *r*_21_ = 1.6 μm, and *r*_22_ = 1 μm.

Figure 1 depicts the unit cell structure of the proposed terahertz perfect absorber. The structure consists of five distinct layers, which is a common configuration. The bottom layer is a gold substrate with a thickness of 0.2 μm, which is utilized to completely reflect the incident terahertz waves. Owing to its high electrical conductivity of 4.7 × 10^7^ S/m, the gold ground plane functions as a mirror, effectively blocking transmission. This is because its skin depth (*δ* = 2/*ωμσ* = 0.13 μm) terahertz band is less than the specified thickness, resulting in zero transmission. The middle layer is a silicon dioxide (SiO_2_, relative permittivity *ε* = 3.9) dielectric layer with a thickness of 1.8 μm; the top layer consists of four patterned single-layer square graphene patches with a default Fermi level of 1 eV. Above the graphene is a thin layer of SiO_2_ used to isolate the metal rings from graphene. This prevents metal rings from generating electric currents due to their high conductivity when different regions of graphene have varying Fermi levels, which could otherwise affect device performance. At the top of the device, there are two sets of concentric gold rings. Here, the inner ring is referred to as *r*_2_, and the outer ring as r_1_. The structure is arranged periodically with a lattice constant of 6 μm. It is noticeable that we divided the graphene layer into four independent regions (G1, G2, G3, G4). Each region’s Fermi level can be tuned individually by biasing with a different voltage through independent electrodes, which is the foundation of realizing logical functions. The simulation of the entire structure is conducted using the Finite-Difference Time-Domain method (FDTD). To simulate the optical response of an infinite periodic array, we applied periodic boundary conditions in the x and y directions [40,41]. In the z-direction, i.e., the direction of light propagation, we used a perfectly matched layer to simulate open space. This setup allowed us to calculate the reflection spectrum R(*ω*) and transmission spectrum T(*ω*) of the structure, and subsequently obtain the absorption spectrum via the formula A(*ω*) = 1 − R(*ω*) − T(*ω*).

Graphene is selected as the tunable material, primarily due to its combined advantages in the terahertz band: efficient electrical tunability, low-loss surface plasmon response, and two-dimensional structural benefits. Its Fermi level can be continuously tuned on a picosecond timescale via gating, providing the physical basis for dynamically controlling absorption peaks. Meanwhile, the high-Q plasmon modes supported by graphene facilitate strong field localization, which is advantageous for narrowband resonance and sensing. Furthermore, its atomic thickness allows seamless integration into multilayer film stacks without disrupting longitudinal resonant modes.

### 2.2. Electrical Properties of Graphene

The surface conductivity *σ_g_* of graphene can be described by the Kubo formula, which consists of contributions from intra-band and inter-band transitions [42].

(1) $$\sigma_{g}\left(\omega ,\mu_{c},\tau ,T\right)={\;\sigma }_{\mathrm{inter}}+{\;\sigma }_{\mathrm{intra}}$$

(2) $$\sigma_{\mathrm{inter}\;}=\frac{ie^{2}}{4\pi \hbar }\ln\left[\frac{2\left|\mu_{c}\right|\;-\;\left(\omega +i\tau^{-1}\right)\hbar }{2\left|\mu_{c}\right|+\left(\omega +i\tau^{-1}\right)\hbar }\right]\;$$

(3) $$\sigma_{\mathrm{intra}}=\frac{ie^{2}k_{B}T}{\pi \hbar^{2}\left(\omega +i\tau^{-1}\right)}\left[\frac{\mu_{c}}{k_{B}T}+2\ln\left(e^{-\frac{\mu_{c}}{k_{B}T}}+1\right)\right]$$

In the formula, *μ_c_* represents the chemical potential. The chemical potential *μ_c_* is not fixed, but is dynamically tuned by an external gate voltage *V_g_*. Based on the parallel-plate capacitor model, the induced carrier sheet density n is given by the following:

(4) $$n=\frac{C_{g}(V_{g}-V_{D})}{e}\;,\;C_{g}=\frac{\varepsilon_{0}\varepsilon_{r}}{d}\;$$

where *C_g_* is the gate capacitance per unit area, *V_D_* is the Dirac point voltage, and d is the dielectric thickness. Owing to graphene’s linear dispersion relation *E*(*k*) = *ħv_F_*|*k*|, the chemical potential shifts with voltage, as follows [43]:

(5) $$\mu_{c}(V_{g})=\hbar v_{F}\sqrt{\pi |n|}sgn(n)=\hbar v_{F}\sqrt{\frac{\pi C_{g}|V_{g}-V_{D}|}{e}}sgn(V_{g}-V_{D})\;$$

In our study, the Fermi level of graphene is set between 0.6 eV and 1.0 eV, while the operating frequency band is 35–55 THz, corresponding to a photon energy of *ħω* ≈ 0.14–0.22 eV. This satisfies a crucial physical condition, 2*Ef* >> *ħω*, meaning the incident photon energy is much smaller than twice the Fermi level. Under this condition, inter-band transitions are strongly suppressed by the Pauli exclusion principle, as the energy of the required final state for an electron to transition from the valence band to the conduction band is much higher than *ħω*, resulting in an extremely low transition probability [44,45]. Therefore, the contribution of the inter-band transition term in the Kubo formula becomes negligible and can be omitted. The model simplifies to the Drude model [46].

(6) $$\sigma_{g}\left(\omega \right)=\frac{ie^{2}\mu_{c}}{\pi \hbar^{2}\left(\omega +i\tau^{-1}\right)}$$

(7) $$\tau =\frac{\mu \mu_{c}}{eV_{f}^{2}}\;$$

In the formula, *e* represents the elementary charge, *ħ* is the reduced Planck constant, *ω* denotes the angular frequency, and *τ* stands for the carrier relaxation time, which is related to carrier mobility *μ* and Fermi velocity *V_f_*. For the purposes of this work, we have chosen *μ* = 1 × 10^4^ cm^2^/V·s and *V_f_* = 1 × 10^6^ m/s.

In this structure, regional tuning of the graphene Fermi level is achieved through an independent embedded electrode configuration. By applying a local bias voltage *V_g_* between a specific graphene region and the back gate, a vertical electric field is formed within that region, dynamically adjusting its Fermi level. This design enables independent control of the four logic input ports [47].

## 3. Results and Discussion

### 3.1. Analysis of the Three-Peak Perfect Absorber

**Figure 2:**
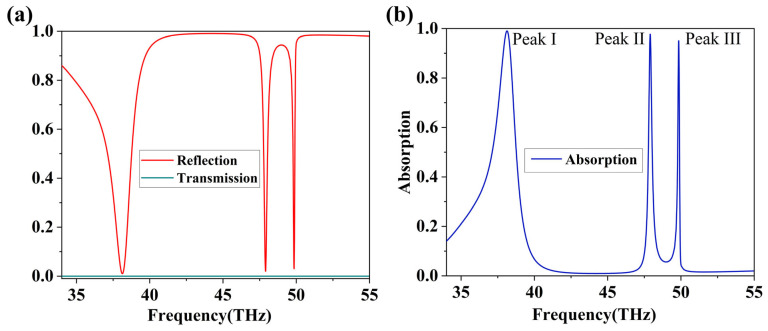
(**a**) Reflection and transmission spectra of the device. (**b**) Absorption spectra of the device.

**Figure 3:**
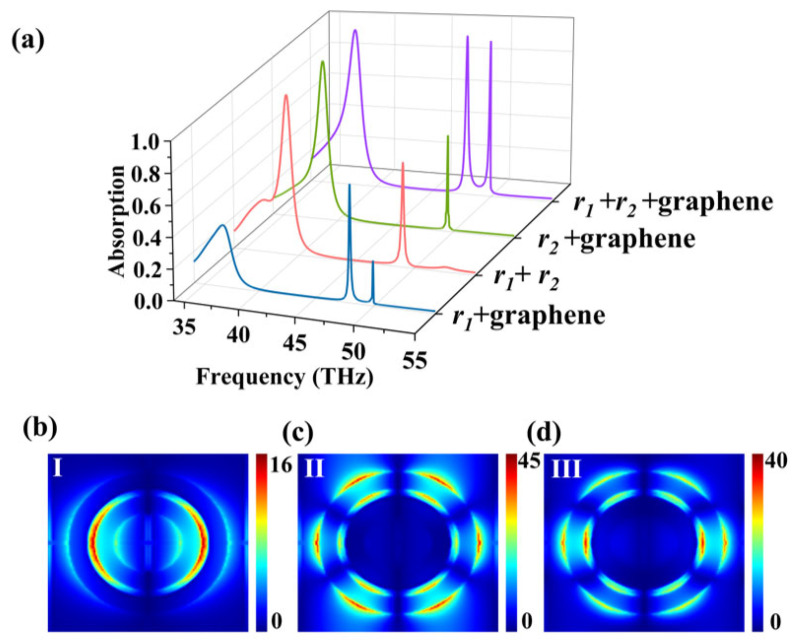
(**a**) Absorption spectra of the device under different structures. (**b**–**d**) Electric field diagrams of Peak I, Peak II, and Peak III in graphene layer.

We first study the absorption performance of the entire structure under normal incidence of terahertz waves. The absorption rate A(*ω*) is determined using the formula A(*ω*) = 1 − R(*ω*) − T(*ω*). The gold substrate is thick enough, T(*ω*) = 0, so A(*ω*) = 1 − R(*ω*). In Figure 2b, the blue solid line displays the absorption spectrum of the complete structure, where we can observe clearly three visible absorption peaks: a relatively broad absorption peak (Peak I) with a peak absorptance of 99.05% at 38.128 THz, and two extremely narrow absorption peaks (Peak II and Peak III) at 47.909 THz (99.56% absorption) and 49.873 THz (97.24% absorption), respectively. In order to understand the physical origin of the three absorption peaks, we respectively simulated the absorption spectra of their substructures, consisting of graphene in the outer ring, graphene in the inner ring, and double-metal ring without graphene. Figure 3a presents the absorption spectra of different structural configurations. The image clearly demonstrates that each component is indispensable for achieving perfect triple-band absorption; perfect three-peak absorption only manifests in the “Graphene + Dual-Ring” structure. From the figure, it can be observed that Peak I is a relatively broad absorption peak, a typical low-Q characteristic of Fabry–Pérot cavity resonance [48], in sharp contrast to the sharp, narrow peaks based on localized surface plasmons, such as Peaks II and III. As shown in Figure 3a, Peak I is strongly present in the “Inner Ring + Graphene” and “Pure Double-Ring” substructures, but nearly absent in the “Outer Ring + Graphene” substructure. This indicates that the generation of Peak I relies on a multilayer film structure comprising the outer ring, graphene, and the bottom gold backplane, featuring well-defined upper and lower reflective surfaces—a classic configuration for a Fabry–Pérot cavity. Furthermore, it can be observed that Peaks II and III only exist when both graphene and the outer ring (*r*_1_) are simultaneously present. This elucidates the dependence of absorption Peaks II and III on both graphene and the outer ring, providing strong supporting evidence for our subsequent analysis in the electric field distribution figures. Figure 3 depicts that the complicated coupling and hybridization between graphene and the localized surface plasmon modes of the two metallic rings result in three peaks in the complete structure. Figure 3b–d display the distribution of the electric field corresponding to the three absorption peaks in Figure 2b. It intuitively shows the different modes of excitation and coupling. The electric field distribution of Peak I is primarily concentrated between inner and outer rings, showing that it stems from a broad-area Fabry–Pérot cavity resonance formed by the whole structure. The core of the Fabry–Pérot resonance is the resonant cavity constituted by the upper and lower surfaces of the silicon dioxide layer. Its resonance condition is determined by the thickness and refractive index of the central dielectric layer, as well as the electrical properties of graphene. Since the resonance is predominantly governed by the optical properties of the dielectric layer, the influence of graphene’s electrical characteristics on absorption Peak I is thereby diminished. Consequently, Peak I exhibits insensitivity to changes in the localized electrical properties of the graphene regions. The electric field of Peak II is highly localized at the outer ring, and its distribution exhibits hexagonal symmetry. This symmetry primarily stems from a high-order localized surface plasmon resonance (LSPR) [49] mode supported by the circular metal ring resonator. Analysis indicates that Peak II corresponds to a mode with an azimuthal mode number of m = 3. The azimuthal distribution function of its electric field intensity |E| takes the form |cos(3*φ*)|, which results in six equally spaced electric field maxima [50], appearing as hexagonal symmetry in the figure. The role of the incident linearly polarized light is to effectively excite this specific mode and determine its fixed orientation in space. This phenomenon reveals that Peak II is not a simple dipole resonance, but rather a higher-order resonance with a more complex mode shape. When the graphene structure is removed, leaving only the double gold ring structure, it can be observed that the position of Peak II is 48.4 THz, which is the intrinsic frequency of the gold rings’ effect. Upon the addition of graphene, a slight shift of the peak to 47.9 THz can be observed. This demonstrates that the frequency of Peak II is primarily determined by the intrinsic frequency of the outer ring LSPR, with slight perturbation due to modulation by the dielectric environment of graphene. The electric field distribution of Peak III is key to understanding its unique working principle. The field of Peak III exhibits strong localization at the outer ring and extends significantly into the graphene plane beneath the inner ring. Furthermore, as can be seen from Figure 3a, Peak III is only significantly present in the “complete structure” and the “outer ring + graphene” substructure, while it almost disappears in the “inner ring + graphene” and “pure double-ring” substructures. This strongly proves that the generation of Peak III necessitates the simultaneous presence of two elements: the outer metal ring and graphene. They are the two fundamental resonators forming this resonance. This indicates that Peak III is a hybrid mode formed by strong coupling between the outer ring LSPR and graphene.

### 3.2. Varying and Analysis of Structural Parameters

**Figure 4:**
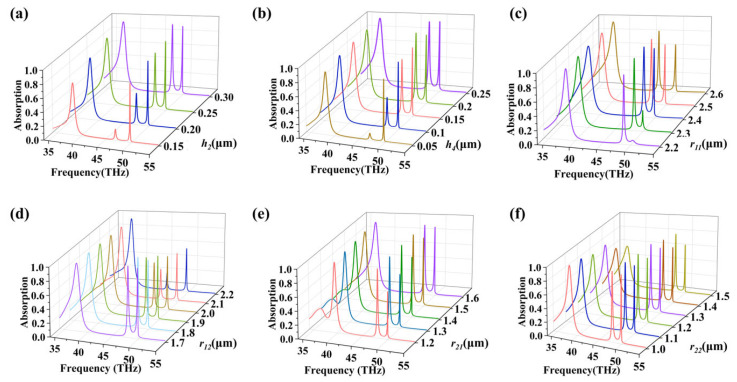
(**a**–**f**) Absorption spectra corresponding to different values of *h*_2_, *h*_4_, *r*_11_, *r*_12_, *r*_21_, and *r*_22_.

In order to maximize the absorption rate and analyze the influence of the absorber’s parameters on the absorption rate, we systematically vary the pivotal structural parameters, including the thickness of metal rings *h*_4_, the thickness of SiO_2_ dielectric layer *h*_2_, the inner and outer radii of outer metal rings *r*_11_ and *r*_12_, and the inner and outer radii of inner metal rings *r*_21_ and *r*_22_. Figure 4 illustrates the modulating effects of key geometric parameters on the absorption spectrum. It should be clarified that the frequency and intensity of Peak I are primarily and significantly influenced by the thickness of the dielectric layer (*h*_4_), while they are relatively insensitive to variations in the fine geometric dimensions of the metal rings. As shown in Figure 4a, which depicts the change in absorption rate with the variation in the metal ring thickness *h*_4_, we can observe that the absorption at Peak II increases strikingly as the thickness of the gold ring increases, reaching its maximum absorption rate when the gold ring thickness is 0.2 µm. Figure 4b shows the change in absorption rate with the variation in the SiO_2_ dielectric layer thickness *h*_2_. The absorption at Peak II increases markedly with the increasing thickness of the SiO_2_ layer, reaching its maximum absorption rate when the SiO_2_ thickness is 0.3 µm. Figure 4c exhibits the change in absorption rate with the variation in *r*_11_. The absorption at Peak III first increases and then decreases as *r*_11_ increases, reaching its maximum absorption rate when *r*_11_ is 2.4 µm. Figure 4d describes the change in absorption rate with the variation in *r*_12_. The absorption rates at both Peak I and Peak II decrease signally as *r*_12_ increases, reaching their maxima when *r*_12_ is 1.7 µm. Figure 4e portrays the change in absorption rate with the variation in *r*_21_. The absorption at Peak III increases with the increasing *r*_21_, reaching its maximum when *r*_21_ is 1.6 µm. Figure 4f represents the change in absorption rate with the variation in *r*_22_. The absorption rate at Peak I decreases as *r*_22_ increases, achieving its maximum when the *r*_22_ is 1.0 µm. By optimizing, we determine the structural parameters, so that we can achieve optimum triple-band perfect absorption. By varying the structural parameters, we can cognize that the three peaks are not simply formed by the coupling between a single ring and graphene, but are the outcomes of interactions among assorted structures.

**Figure 5:**
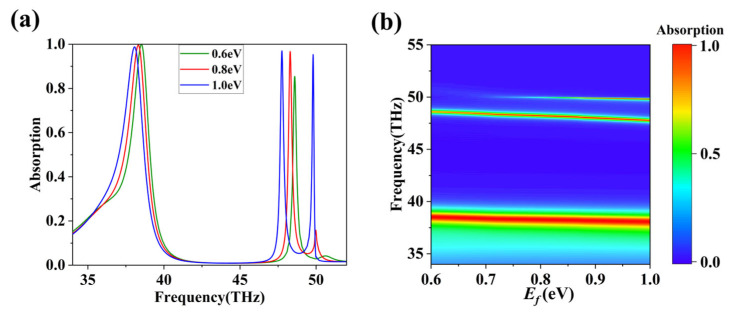
(**a**) Absorption spectra associated with varying Fermi levels of graphene. (**b**) Relationship between absorption spectra of the absorber and Fermi level ranging from 0.6 eV to 1.0 eV.

We investigated the variations in the spectral positions of the absorption peaks in response to changes in the Fermi level. The absorption curves in Figure 5 clearly show significant changes in the spectra as the Fermi level increases from 0.6 eV to 1.0 eV. First, the absorption peaks undergo a blue shift. With the rise of *E_f_*, all three absorption peaks systematically move towards lower frequencies. When the Fermi level increases from 0.6 eV to 1.0 eV, Peak I shifts from approximately 38.5173 THz to about 38.0025 THz; Peak II shifts from approximately 48.5918 THz to about 47.5728 THz; and Peak III shifts from approximately 50.7244 THz to about 49.7369 THz. Secondly, the absorption rate changes. The peak value of Peak III decreases remarkably as the Fermi level decreases. At a Fermi level of 1.0 eV, the absorption rate peaks at 98%. However, when the Fermi level decreases to 0.8 eV, the absorption rate declines to 20%, and further reduction to 0.6 eV results in an absorption rate of only 3.5%. This implies that Peak III is extremely sensitive to changes in the Fermi level. This unique sensitivity can be utilized for the fabrication of logic gates.

### 3.3. The Four-Input AND Logic Gate

**Figure 6:**
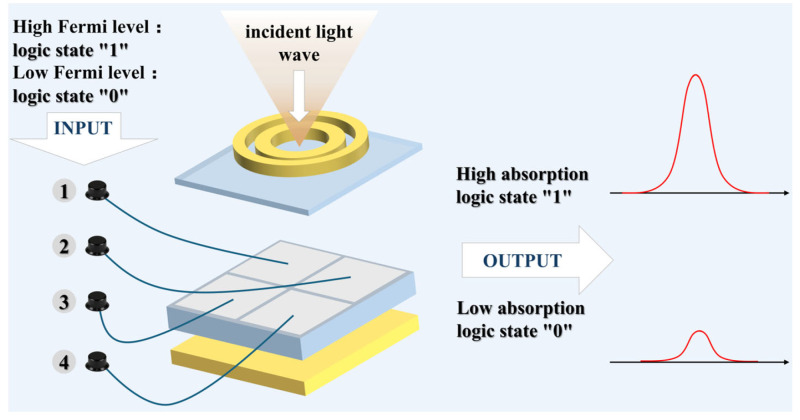
Illustration depicting the operational principle of a four-input AND logic gate.

**Figure 7:**
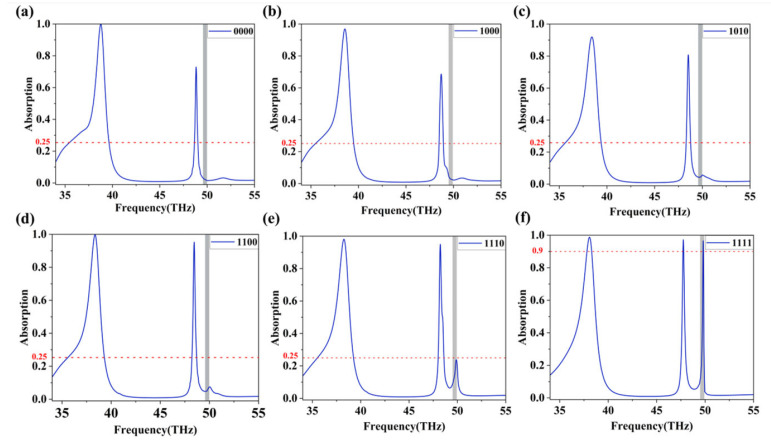
Four-input AND gate at 49.873 THz. (**a**) G1 = G2 = G3 = G4 = 0.3 eV. (**b**) G1 = 1.0 eV, G2 = G3 = G4 = 0.3 eV. (**c**) G1 = G4 = 1.0 eV, G2 = G3 = 0.3 eV. (**d**) G1 = G2 = 1.0 eV, G3 = G4 = 0.3 eV. (**e**) G1 = G2 = G3 = 1.0 eV, G4 = 0.3 eV. (**f**) G1 = G2 = G3 = G4 = 1.0 eV.

The tunable graphene Fermi level enables dynamic control of absorption characteristics. We found that Peak III, at 49.873 THz, is extremely sensitive to the changes in Fermi level of the graphene region directly beneath it. When the Fermi level of graphene is reduced, the positions of all three peaks undergo slight blueshifts. However, the absorption rates of Peaks I and II show minimal change, whereas the absorption of Peak III exhibits a significant variation. Based on this phenomenon, we designed a four-input AND logic gate. The working principle of the logic gate is depicted in Figure 6. We regard four separate graphene regions (G1–G4) as the four logical input ports. A Fermi level of 0.3 eV in a region is defined as the “low” state, and the input logic is “0”. A Fermi level of 1.0 eV in a region is defined as the “high” state, and the input logic is “1”. The logical output is defined as the absorption intensity at 49.873 THz frequency. Therefore, we set a threshold: an absorption rate more than 90% represents logic“1”, while an absorption rate less than 25% represents logic“0”. The structure consists of four graphene regions, namely, four input ports. Each port has two states: “1” and “0”, so there are 16 possible input combinations. The 16 cases of this logic gate are presented in Table 1. Due to the periodic structure we designed, when only one region is in the “1” state, its specific location does not influence the outcome. Likewise, when only one region is in the “0” state, its location also has no impact. When both inputs are “1”, we analyze two scenarios: one with the two “1” inputs adjacent, and the other with them diagonal. Thus, Figure 7 presents output outcome under six different input combinations. Only when all four inputs are “1”, meaning the Fermi levels of G1 to G4 are all 1 eV, can Peak III maintain a high absorption rate, resulting in an output of “1”. As long as any input is “0”, the absorption rate of Peak III sharply declines below the threshold, resulting in an output of “0”. This perfectly realizes the AND logic function. Figure 7 displays the absorption spectra of the absorber, which correspond to the six different input types. As shown, a high absorption rate (>90%) is achieved exclusively under the condition where all four inputs are “1”. For any other input combination, including three, two, one, or no “1”s, the absorption rate stays below 25%, thereby perfectly emulating the characteristic response of a four-input AND logic gate.

The generation mechanism of Peak III is a hybrid mode formed by the strong coupling between the outer ring LSPR and graphene. Its resonant characteristics are primarily determined by the geometric symmetry of the outer ring and the overall electrical environment of graphene. Even in the presence of local differences in the Fermi level (*Ef*), the polarization dependence introduced by bias asymmetry is weak, due to the rotational symmetry of the outer ring itself and the fact that the coupling involves the collective response of graphene. Meanwhile, the core criterion for the logic gate function is “whether the absorption rate of Peak III exceeds the threshold of 90% or falls below 25%.” The logic gate’s output yields a high absorption rate only when all four inputs are 1. Therefore, as long as we ensure that the difference between the condition where all four inputs are 1 and all other conditions is measurable, the logic function can be reliably realized. The simulation results show that, only when all inputs are 1 is the absorption rate greater than 90%, while, in all other cases, it is below 25%. The gap between these states is substantial and, thus, can be easily measured.

To demonstrate that the metamaterial absorber possesses digital information processing capabilities beyond traditional analog modulation, we directly embedded Boolean logic functionality into this physical system by encoding the electrical states (Fermi levels) of the four graphene regions as binary logic inputs and mapping the absorption state of Peak III to the logical output. This successfully realized a functional four-input AND logic gate, marking the evolution of the device from a passive analog response unit to an active digital functional unit.

### 3.4. High-Sensitivity Sensing Performance

**Figure 8:**
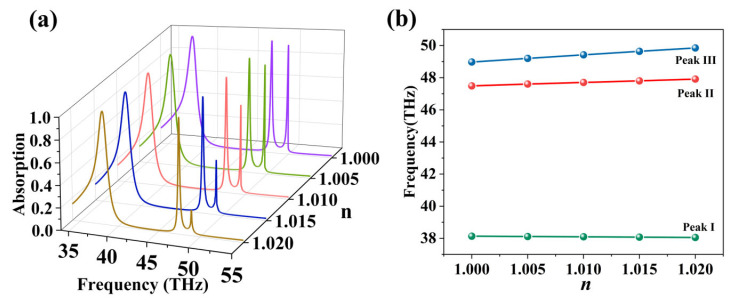
(**a**) Variations in absorption spectra during the ambient refractive index rises from 1 to 1.02. (**b**) Variation curves of peak positions for Peak I, Peak II, and Peak III during the ambient refractive index rise from 1 to 1.02.

Besides electrical tuning characteristics, we also investigated the performance of the structure serving as a refractive index sensor. The displacements of the absorption peaks are monitored by varying the refractive index of the analyte on the structure’s surface. As shown in Figure 8, with the ambient refractive index increasing from 1.0 to 1.02, the two narrow peaks (Peak II and Peak III) underwent obvious red shift, while keeping excellent line shapes. The sensitivity (*S*) is characterized by the ratio of the resonance peak frequency shift (Δ*f*) to the change in the ambient refractive index (Δ*n*):

(8) $$S\;=\;\frac{\Delta f}{\Delta n}$$

Via linear fitting, we calculated the sensitivity values of Peak II and Peak III at 30.1 THz/RIU and 62.5 THz/RIU, respectively. Furthermore, figure of merit (FOM) serves as another key metric for evaluating the sensor’s performance. FWHM is the full width at half maximum.

(9) $$\mathrm{FOM}=\frac{S}{\mathrm{FWHM}}$$

The FOM values of the two peaks reached 215 and 358, respectively, implying their extraordinary sensing precision and detection capabilities.

One of the core innovations of this work is the integration of tunable absorption, logic operation, and sensing functionalities within a single structure. The three absorption peaks exhibit complementary and synergistic functions and performance: Peak I (broadband, high absorption) primarily serves the function of efficient energy harvesting, suitable for terahertz radiation detection and energy capture; Peak II (sensitivity 30.1 THz/RIU) possesses medium–high sensitivity and high-Q characteristics, acting as an auxiliary sensing channel for environmental interference suppression, cross-validation, or specific analyte identification, thereby enhancing the system’s robustness and information dimensionality; Peak III (sensitivity 62.5 THz/RIU), with its ultra-high peak sensitivity, is specifically dedicated to the detection of high-precision, minute refractive index changes and serves as the core physical carrier for determining the “on/off” states in logic operations. Peak III can be used to detect subtle physicochemical changes in sensing applications [51,52,53], with Peak II serving as an auxiliary reference. The device holds potential for application in the sensor domain. This multi-band collaborative architecture of “broadband energy harvesting, medium-high sensitivity auxiliary sensing, ultra-high sensitivity core sensing/logic” not only ensures the independence of each function, but also enhances the device’s adaptability and functional integration in complex environments.

### 3.5. Polarization and Insensitivity of Incident Angle

**Figure 9:**
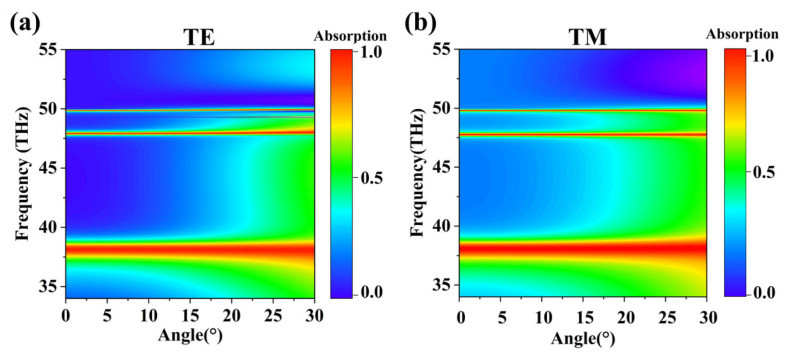
(**a**) The varying diagram of TE wave. (**b**) The varying diagram of TM wave.

The terahertz perfect absorption designed in this paper exhibits marvelous practical performance, and one of its most salient characteristics is its highly stable optical response across a broad spectrum of incident angles [54]. As depicted in Figure 9, for incident angles spanning from normal incidence (0°) to oblique incidence (30°), the multiple resonance absorption peaks achieved by the absorber retain superb stability in frequency and absorption rate. Furthermore, the remarkable stability extends to incident light of both TE and TM polarizations, highlighting the device’s inherent polarization-insensitive nature. This characteristic allows it to effectively operate under diverse and complex real-world illumination scenarios. This wide-angle operational ability and polarization-insensitive property are key indices to evaluate the practical values of metamaterial absorbers, meaning that, in practical applications such as terahertz imaging, non-destruction tests, high-speed communication, and energy collection, the device can sharply decrease the stringent alignment precision requirements of optical systems and maintain highly reliable and accordant performance under the complex or changing illumination conditions. This simplifies the complexity of system integration and reduces the cost, as well as markedly enhancing the robustness and practical potential of the device in the real environment, laying a solid foundation for its transition to practical applications (Table 2).

## 4. Conclusions

This article details the successful design and theoretical research of a tunable triple-peak perfect absorber based on a graphene–metal ring composite structure. The structure achieves triple-band perfect absorption through a combination of broad and narrow bands. The physical mechanisms correspond respectively to Fabry–Pérot cavity resonance and the coupling between the localized surface plasmons of the outer ring and graphene surface plasmon polaritons. It also fully exploits the electrical tunability of graphene to realize a four-input AND logic gate function. The absorption rate of the absorber is taken as the output, with the Fermi level of graphene serving as the input, and a four-input AND gate capable of performing logical operations is achieved. This design combines traditional absorption functionality with logic operations, offering broad prospects for terahertz information and communication, especially in the realization of logic functions. Furthermore, the structure exhibits high-sensitivity refractive index sensing capability. Through parameter varying, the structural performance is optimized, and its polarization and incident angle stability are proved. This study provides a valuable design paradigm and theoretical reference for realizing reconfigurable terahertz photonic devices that integrate dynamic modulation, logic operations, and sensing. It holds broad application prospects in areas such as terahertz communication encryption, intelligent environmental perception, and sensing–computing integrated systems.

## Figures and Tables

**Table 1:** Validation table for the four-input AND logic function.

Input (G1, G2, G3, G4)	Logical State	Absorption (49.873 THz)	Output
(0, 0, 0, 0)	0000	<25%	0
(1, 0, 0, 0)	1000/0100/0010/0001	<25%	0
(1, 1, 0, 0)	1100/0011/1010/0101	<25%	0
(1, 0, 0, 1)	1001/0110	<25%	0
(1, 1, 1, 0)	1110/1101/1011/0111	<25%	0
(1, 1, 1, 1)	1111	>90%	1

**Table 2:** Comparison of this work with other works.

Feature	Logic Function	Sensing Sensitivity	Absorption Peaks	Tuning Mechanism
[55]	None	0.4 THz/RIU	Broadband	Structural
[56]	Single-input NOT gate	Not specified	Multi-band	Graphene
[57]	None	3.6 THz/RIU	5 Peaks	Graphene
This work	4-input AND gate	62.5 THz/RIU	3 Peaks	Graphene

## Data Availability

Data underlying the results presented in this paper are not publicly available at this time, but may be obtained from the authors upon reasonable request.
